# Correction of motion‐induced misalignment in co‐registered PET/CT and MRI (T1/T2/FLAIR) head images for stereotactic radiosurgery†

**DOI:** 10.1120/jacmp.v12i1.3306

**Published:** 2010-10-07

**Authors:** Guang Li, Huchen Xie, Holly Ning, Deborah Citrin, Aradhana Kaushal, Kevin Camphausen, Robert W. Miller

**Affiliations:** ^1^ Radiation Oncology Branch, Center for Clinical Research National Cancer Institute, National Institutes of Health Bethesda MD 20892 USA

**Keywords:** 3D volumetric image registration, co‐registered PET/CT and MRI/MRI images, head motion, target delineation and localization, high‐precision stereotactic radiosurgery (SRS)

## Abstract

The purpose was to evaluate and correct the co‐registration of diagnostic PET/CT and MRI/MRI images for stereotactic radiosurgery (SRS) using 3D volumetric image registration (3DVIR). The 3DVIR utilizes the homogeneity of color distribution over a volumetric anatomical landmark as the registration criterion with submillimeter accuracy. Fifty‐three PET/CT and MRI (T1, T2 and FLAIR) image sets of patients with brain lesions were acquired sequentially from a hybrid PET/CT or an MRI scanner with common diagnostic head holding devices. Twenty‐five sets of head ${\;}^{18}F-\text{FDG}-\text{PET}/\text{CT}$ images were scanned over a 10‐minute interval and 14 whole‐body sets were scanned over a 30‐minute interval. Fourteen sets of MRI images were acquired, and each 3‐modal image set (T1, T2 and FLAIR) was scanned in sequence at time 0, ~5 and ~20 minutes. The misalignments in these “co‐registered” images were evaluated and corrected using the 3DVIR. Using the head immobilization devices commonly found in diagnostic PET/CT and MRI/MRI imaging, 80%–100% of these “co‐registered” images were identified as misaligned. For PET/CT, the magnitude of misalignment was 0.4°±0.5° and 0.7±0.4mm for 10‐minute scans, and 0.8°±1.2° and 2.7±1.7mm for 30‐minute scans. For MRI/MRI, the magnitude was 0.2°±0.4° and 0.3±0.2mm for 5‐minute scan intervals, and 1.1°±0.7° and 1.2±1.4mm for 20‐minute intervals. Small, but significant, misalignment is present in the co‐registered diagnostic PET/CT and MRI/MRI images and can be corrected in SRS treatment planning using the volumetric image registration for improved target localization within the clinical error tolerance.

PACS numbers: 87.53.Ly, 87.57.nj, 87.57.uk, 87.57.Q‐, 87.61.jc, 87.19.xc

Conflict of Interest Statement: The authors do not have any conflict of interest on this research report.

## I. INTRODUCTION

Biological imaging, such as positron emission tomography (PET), has been increasingly applied to radiation treatment planning (RTP) for target delineation, aiming to improve the outcome of local control.^(^ ^1^ ^–^ ^5^ ^)^ Hybrid scanners combining PET and computed tomography (CT) are utilized to provide inherently “co‐registered” functional and anatomical image sets.^(^ ^6^ ^)^ This, however, assumes that during image acquisition the patient remains motionless, which is often violated under clinical conditions. Accurate PET/CT image alignment is essential for precise delineation and localization of the gross target volume (GTV).^(^ ^7^ ^,^ ^8^ ^)^ In addition, magnetic resonance imaging (MRI) provides high soft tissue contrast and is often required for target delineation,^(^ ^9^ ^,^ ^10^ ^)^ especially for stereotactic radiosurgery (SRS) procedures. Different MRI sequences, such as T1, T2 or fluid attenuated inversion recovery (FLAIR), enhance different aspects of soft tissue, helping to distinguish the GTV from local edema. In brain cancer^(^ ^3^ ^)^ and recently nasopharyngeal cancer,^(^ ^11^ ^)^ combining PET/CT image with MRI image for SRS target delineation shows promise. The clinical tolerance for target localization, including image registration, should be within 1 mm for SRS.^(^ ^3^ ^,^ ^11^ ^–^ ^14^ ^)^ Therefore, the uncertainty of multimodality image registration must be well below this tolerance in order to ensure a successful SRS treatment.

Although the combined PET/CT scanner or sequential MR imaging can produce co‐registered images,^(^ ^15^ ^)^ the patient immobilization device commonly used for diagnostic imaging may not provide the high degree of accuracy required for a high‐precision therapeutic procedure, such as SRS. A quantitative evaluation on four different head holding devices was reported, based on various head motions observed using infrared motion tracking camera.^(^ ^16^ ^)^ Even the best devices permit residual motions on the orders of 1.4 mm/0.3° for a ball‐shaped head holder with small vacuum‐lock bag insert and 2.4 mm/0.4° for a well‐shaped head holder with one‐inch thick construction‐foam insert. To minimize head motion, an invasive stereotactic head frame has been used for CT, MRI and PET/CT imaging to ensure a motionless patient for accurate target localization.^(^ ^17^ ^,^ ^18^ ^)^ However, a special MRI‐compatible frame is required and a well‐coordinated scheduling must be designated, in order to have both the stereotactic imaging (PET/CT, MRI, and CT) and treatment (SRS) in the same day. Without using the stereotactic frame, the assumed co‐registration is carried over clinically from registration of the CT in PET/CT to the planning CT, or from the registration of one of the sequential MRI (T1, T2 and FLAIR) images to the planning CT. Thus, correction for any potential misalignment in nonstereotactic imaging is necessary to achieve the SRS accuracy (~1.0 mm).

It is challenging to register PET/CT images using software and to visually verify the alignment, due to lack of complete anatomy in PET images and low spatial resolution. Automatic registration using mutual information may improve the alignment but it requires visual verification, and the orthogonal planar (2D) views are not effective to handle the anatomy‐deficient, low‐resolution PET image. Even for anatomic image registration, the conventional 2D‐based fusion can suffer from low precision,^(^ ^19^ ^)^ large interobserver variability,^(^ ^20^ ^)^ as well as unrealized registration error.^(^ ^21^ ^)^ Some commercial fusion software does not easily allow re‐alignment of the co‐registered images. Recently, 3D volumetric image registration (3DVIR) has been developed for multimodality registration of CT, MRI and PET images with sub‐mm accuracy.^(^ ^21^ ^–^ ^22^ ^)^ This volume‐view–guided image registration employs the homogeneity of color distribution over a volumetric anatomical landmark as the registration criterion, which provides an extra dimension beyond the volumetric anatomy. At a high‐contrast anatomical interface, such as skin and air or bone and soft tissue, a fractional voxel misalignment can be amplified with a dramatic visual effect as of color inhomogeneity.^(^ ^22^ ^)^ Therefore, the 3DVIR is capable of detecting and correcting subtle misalignment in co‐registered PET/CT and MRI/MRI head images.

In this study, the alignment of co‐registered PET/CT and MRI (T1/T2/FLAIR) images of 53 patients was evaluated using the 3DVIR. Misalignments were found in more than 80% of these image sets, and the amplitudes were consistent with previously reported data based on optical measurements using a similar diagnostic immobilization device.^(^ ^16^ ^)^ The misalignments can and should be corrected for better target localization in cranial and nasopharyngeal SRS treatment planning.

## II. MATERIALS AND METHODS

### A. 3D volumetric image registration technique

The 3D volumetric image registration (3DVIR) is a visual‐based manual registration tool to align up to four monocolored multimodality images based on the color homogeneity distributed over a volumetric anatomical landmark.^(^ ^21^ ^)^ Using this criterion, evaluation of the current image alignment and guidance for refinement can be achieved. The detection limit and accuracy of the 3DVIR was shown to be 0.1° and 0.1 voxel (~0.1 mm) for registration of PET/CT, MRI/MRI and CT/CT head phantom images.^(^ ^22^ ^)^ For patient's head images, the accuracy may be reduced, but within 0.5 mm should be readily achievable, since mild local skin deformation, if identified, could be ignored in the 3DVIR and the registration could be verified using another independent landmark, such as the brain.

An opacity parameter (alpha) in RGBA (red, green, blue and alpha) voxel format^(^ ^23^ ^,^ ^24^ ^)^ was used to view internal anatomies through manipulation of a window/level (W/L, or a simplified form of lookup table, LUT) to control the transparency over the image histogram, as shown in Figure 1. The CT, MRI and PET images were assigned a color and rendered in real time using a VolumePro (TeraRecon, Inc., San Mateo, CA) video card to serve as a sophisticated graphic processing unit (GPU) in a PC computer. All images were preprocessed automatically to have an 8‐bit grayscale (one‐fourth of the 32‐bit voxel buffer), 320×320 image size, and isotropic voxel size with trilinear interpolation, after the images were manually adjusted for optimal visualization using the W/L. Detailed description of this 3DVIR technique can be found elsewhere.^(^ ^21^ ^,^ ^22^ ^)^

**Figure 1:**
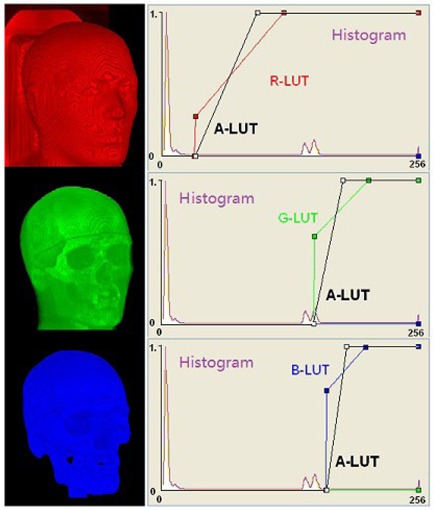
Visualization of volumetric anatomical landmarks in a CT anthropomorphic head phantom using the 3D volumetric image registration (3DVIR). Four lookup tables (LUTs) are assigned to RGBA voxels for control pseudo‐color (RGB) display and transparency (a) of voxels based on the histogram of an image. The transparency LUT (a) is drawn in black.

In this study, the translational coordinate shifts (dXt, dYt, dZt) were defined as the shifts along left‐right, anterior‐posterior, and superior‐inferior directions, respectively. The rotational shifts (dXr, dYr, dZr), however, were defined as the gantry rotation (roll), the couch rotation (yaw), and the tilt rotation (pitch), respectively. The center of rotation was set at the center of the image volume (field of view). After registration, the motion‐corrected images were saved as DICOM series, which possessed the co‐registration status. The alignment of the lesions was visually verified with the conventional three‐orthogonal planar views, particularly for anatomical images, such as postcontrast MRI and CT images. The volumetric registration of multimodality images was straightforward as previously reported.^(^ ^21^ ^,^ ^22^ ^)^

### B. Common volumetric anatomical landmarks for PET, CT and MRI

Anatomical landmarks with high contrast relative to their neighboring voxels are preferable to use, due to their high reliability and simple classification, as well as visual enhancement in the volumetric alignment. In the cranial anatomy, the volumetric skin and brain were employed as registration landmarks, even though these landmarks may not show well in 2D views, as illustrated in Fig. 2. Cross‐verifications using both skin and brain landmarks were performed on some of the registrations. Prior to the PET/CT image alignment, the PET skin volume was adjusted to CT skin by altering the transparency W/L control. Around the nasal‐orbital region with its high degree of curvature, partial volume artifacts due to the low resolution of PET images should be ignored. It was assumed that the patient's head motion reflected a random perturbation around its mean position, so that motion blurring produced minimal effect on the average head position, as well as its volume.

**Figure 2:**
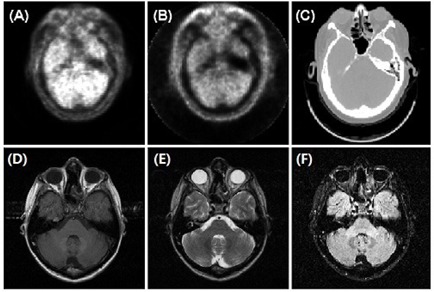
Two‐dimensional views of the common landmark (the skin) for PET with (a) and without (b) attenuation correction, CT (c) and MRI: T1 (d) T2 (e) and FLAIR (f).

### C. Acquisition of patient's head PET/CT and MRI images (T1/T2/FLAIR)

A pool of brain patients with mean age of 49.5±12 years, ranging from 24 to 66 years of age, was included in this retrospective study. All head PET/CT images were acquired from a PET/CT scanner after 60‐minute uptake of 12 mCi of ${\;}^{18}F-\text{fluorodeoxygluco}({\;}^{18}F-\text{FDG})$. The original image sizes of 128×128 (PET) and 512×512 (CT) were converted to 320×320. Twenty‐five PET/CT images were acquired using a scanner (DiscoveryST, GE Healthcare, Waukesha, WI) with two bed positions (10 minutes). A deep U‐shaped head holder with one‐inch thick foam insert was used, as shown in Fig. 3. The final voxel size was $0.80\;{\text{mm}}^{3}$. Fourteen PET/CT head images were taken from whole‐body scans from another PET/CT scanner (Gemini, Siemens Medical Solutions, Malvern, PA), with six bed positions in 30 minutes. The final voxel size was 1.56 mm.

**Figure 3:**
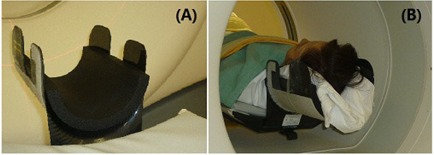
A head immobilization device (U‐shape) used in diagnostic PET/CT imaging: head holder (a) and 1 inch thick foam padding materials; patient setup (b) in the head holder during PET/CT imaging.

Fourteen sets of three sequential images, T1, T2 and FLAIR, were acquired from an MR scanner (Achieva 3T, Philips Medical Systems, Andover, MA). Several foam wedges, together with a head coil frame, were used to restrict head motion. The original MRI image sizes were 512×512 or 256×256, which were converted to 320×320 with the resulting voxel size of $0.69\;{\text{mm}}^{3}$. A set of fiducials was applied in one patient. A full brain MR imaging protocol was applied, in which T1, T2 and FLAIR were acquired in sequence. The T1 and T2 were consecutive scans, about 2–5 minutes apart, while the T1 and FLAIR scans were separated by other scans, about 15–20 minutes apart. Each individual MRI scan may take 2–5 minutes, depending upon the imaging quality requirement.

## III. RESULTS

### A. Correction of misalignment in PET/CT images

Figure 4 (a) and (b) shows an example of the correction of a misaligned PET/CT image using the 3DVIR technique, while Fig. 4 (c) and (d) shows the skin landmarks in both PET and CT. With translational correction of dXt, dYt, and dZt at ‐1.5, ‐1.8, and 0.8 mm, respectively, or an overall displacement of 2.5 mm, the color homogeneity of the skin was improved. The rotational correction was negligible in this case. Figure 5 (a) and (b) shows the average and range of misalignments resulting from two different scan durations. For a 10‐minute acquisition, the average misalignment was 0.4°±0.5° and 0.7±0.4mm, while for the 30‐minute acquisition, the average misalignment increased to 0.8°±1.2° and 2.7±1.7mm. The percentage of misaligned images increased from 88% to 100% and the average amplitude increased by a factor of four as the scanning time was extended. None of the patients had a misalignment greater than 2° or 2 mm in the 10‐minute scans, but these percentages increased from 0% to 14% (>2°) or to 28% (> 2 mm) in the 30‐minute scans.

**Figure 4:**
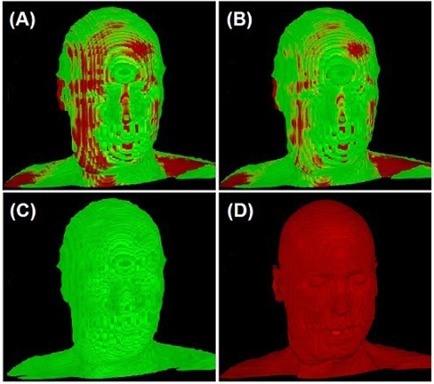
Correction of misalignment in a co‐registered PET/CT image: before (a) and after (b) the 3DVIR correction. The volumetric skin landmark in PET and CT images are shown in (c) and (d), respectively. In PET, the normal tissue uptake of ${\;}^{18}F-\text{FDG}$ can indicate some specific anatomical structures, which were used for registration, as shown in (c). Note that the best volumetric match would show most homogeneity of the color distribution on the skin landmark.

**Figure 5:**
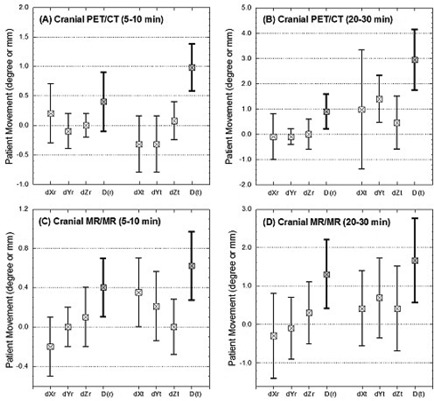
Misalignments in co‐registered PET/CT and MRI/MRI images. The mean and range of head motions are plotted, together with the “distances” of the three translational and rotational displacements. The translations of x, y and z are defined as left‐right, superior‐inferior and anterior‐posterior, respectively. The roll and yaw rotations represent the gantry and couch rotations, respectively, while the pitch corresponds to head nodding action.

### B. Correction of misalignment in MRI/MRI images

Figure 6 shows the correction of misalignment among the co‐registered T1, FLAIR and T2 images, which were acquired with ~5‐ and ~20‐minute intervals. The co‐registration status of T1/FLAIR was confirmed, as shown in Fig. 6 (e) and (f), while the misalignment of the T2 from T1 and FLAIR was corrected. Six external fiducial markers provided an independent check of the registration. The consistency between the intrinsic criteria and extrinsic markers provided a cross‐verification of the 3DVIR registration.

**Figure 6:**
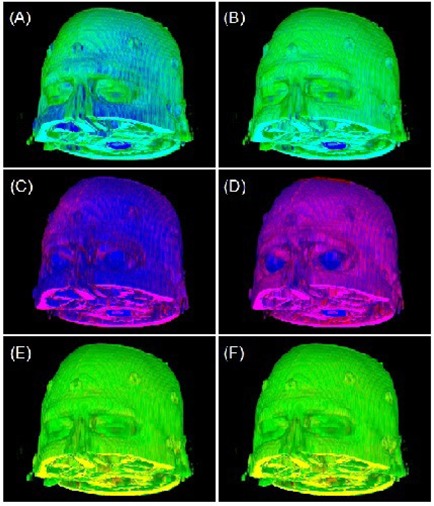
Correction of misalignments among three co‐registered MRI images: T1 (green), T2 (blue), and FLAIR (red). The original images are shown on the left column (Figs. (a), (c) and (e)) and the corrected images are shown on the right column (Figs. (b), (d) and (f)).

Figure 5 (c) and (d) show the average and range of correction for the co‐registered MRI/MRI images. The average misalignment was: 0.2°±0.4° and 0.3±0.3mm for ~5‐minute interval, and 0.8°±1.2° and 2.7±1.7mm for ~20‐minute interval. The percentage of misaligned images increased from 79% to 100% and the amplitude increased by four‐fold, due to the increased acquisition time interval, similar to the result from PET/CT. As the scan time interval increased, the percentage of images that had misalignments greater than 2° or 2 mm increased from 0% to 21% or to 28%, respectively.

## IV. DISCUSSION

### A. Skin landmark for MRI, PET and CT image registration

For rigid anatomy, such as head, the skin is a most convenient and sufficiently reliable landmark. The volumetric skin has advantages over external fiducial markers because the number of available alignment voxel points is substantially greater than the number of fiducials, as shown in Fig. 6. For anatomical (T1, T2 and FLAIR) images, subtle modality differences were observed, as shown in Figs. 2 and 6, and the skin volume can be adjusted using T1 image as a reference.

For functional (PET) images, although the skin is poorly defined in 2D views, as shown in Fig. 2(a), in volumetric views it is anatomically informative, as shown in Fig. 4(c). The brain is another structure that can be utilized. However, PET images cannot be used solely to define the skin volume, as the skin signal is only slightly above the background level and the PET resolution is poor as well. Therefore, CT images were used to define skin volumes, which then served as a reference for defining the PET skin volume.

### B. Correction of misalignment due to voluntary head movements

The holder had a depth of 15 cm and the gap between the head and the holder was filled with a one‐inch foam insert, as shown in Fig. 3. Part of the head immobilization device (around the ear opening) used in this study is shown in Fig. 2(c). This was similar to other head holders reported previously.^(^ ^16^ ^)^ From the 3DVIR correction, the average translational motion (<dT>) increased from 0.5–1 mm to 2–3 mm as the scanning interval increased from 10 to 30 minutes; while rotational variations (<dR>) were approximately 0.5° and 1.0° for these time intervals. These observations are similar to those (~0.5° and ~2 mm) that employed an infrared motion tracking camera system with similar head immobilization.^(^ ^16^ ^)^ The longitudinal translation largely resulted from the couch positioning uncertainty (<1.0 mm), which was detected using the 3DVIR method.^(^ ^22^ ^)^ Lateral motion (dXt) appeared to be the largest translation among the three, while the roll (dXr) was the principal rotational variation, as shown in Fig. 5.

### C. Quality assurance of image registration for stereotactic radiotherapy

The 3DVIR technique can be employed for registration of multimodality images as well as visual alignment verification. As the results show, scanner‐based co‐registration contains high probability of misalignment, which must be corrected for radiosurgery. Although the degree of accuracy (0.1 mm) of 3DVIR was determined from a phantom study,^(^ ^22^ ^)^ the registration uncertainty for cranial images should be less than 0.5 mm, which is sufficiently accurate for SRS. It is worthwhile to mention that deformable image registration may not be useful in tumor alignment. In contrast, the accuracy of 2D‐based visual fusion of CT and MRI images is user‐dependent,^(^ ^19^ ^,^ ^20^ ^)^ and likely contains unrealized errors of exceeding 1 mm.^(^ ^21^ ^)^ For biological PET images, the 2D fusion technique cannot be recommended. Although automatic registration using mutual information can achieve submillimeter registration accuracy, visual verification is required and manual adjustment is often necessary. As a consequence, 2D fusion, which is the only visual tool available clinically, has to be used with an associated higher risk of > 1 mm uncertainty.

The use of the 3DVIR may eliminate the need of an invasive head frame for pretreatment PET/CT and MRI imaging related to SRS.^(^ ^17^ ^,^ ^18^ ^)^ Therefore, diagnostic images can be utilized through volumetric image registration with a level of accuracy sufficient for SRS. In a trend of increasing clinical implementations of image‐guided frameless SRS,^(^ ^25^ ^,^ ^26^ ^)^ where stereotactic imaging is not possible, the 3DVIR could be applied to achieve the accuracy of stereotactic imaging for target delineation and localization, and to perform image‐guided stereotactic setup for frameless SRS delivery.

## V. CONCLUSIONS

Co‐registered PET/CT and MRI/MRI images (80%–100%) contain misalignments (0.5°−1.0° and 1.0−3.0 mm) due to patient motion during 5 to 30 minutes of acquisition using diagnostic head immobilization devices. The 3D volumetric image registration, alone or in combination with automatic registration, provides high level of accuracy and reliability for identifying and correcting the misalignments, and therefore improves target localization in stereotactic radiosurgery.

## ACKNOWLEDGEMENTS

The authors are grateful to Dr. Carolyn Melzer and Ms. Cristy Matan (University of Pittsburgh Medical Center) for kindly providing the whole‐body PET/CT images.
